# β2-Chimaerin Deficiency Favors Polyp Growth in the Colon of Apc^Min/+^ Mice

**DOI:** 10.3390/molecules30040824

**Published:** 2025-02-11

**Authors:** Eladio A. Velasco-Sampedro, Cristina Sánchez-Vicente, María J. Caloca

**Affiliations:** Instituto de Biomedicina y Genética Molecular (IBGM), CSIC-UVA, 47003 Valladolid, Spaincrissv@uva.es (C.S.-V.)

**Keywords:** β2-chimaerin, Rac1, GTPase-activating protein (GAP), Apc, colon cancer, ERK

## Abstract

A Rho-GTPases are pivotal regulators of key cellular processes implicated in colorectal cancer (CRC) progression, yet the roles of their regulatory proteins, particularly GTPase-activating proteins (GAPs), remain poorly understood. This study focuses on β2-chimaerin, a Rac1-specific GAP, in Apc-driven tumorigenesis using the ApcMin/+ mouse model. We demonstrate that β2-chimaerin deficiency selectively promotes the growth of colonic polyps without influencing small intestinal polyp formation. Mechanistically, β2-chimaerin loss is associated with enhanced ERK phosphorylation, while canonical Wnt/β-catenin and E-cadherin pathways remain unaffected, suggesting its specific involvement in modulating proliferative signaling in the colon. Consistent with its tumor-suppressive role, bioinformatics analyses reveal that low β2-chimaerin expression correlates with poor prognosis in CRC patients. This study expands the understanding of Rho-GTPase regulatory mechanisms in intestinal tumorigenesis, providing a basis for future therapeutic strategies targeting Rho-GTPase pathways in CRC.

## 1. Introduction

Colorectal cancer (CRC) is one of the most prevalent cancers, ranking as the third most frequently diagnosed cancer worldwide and the second leading cause of cancer-related death [1]. Despite significant advancements in understanding the molecular basis of CRC, the high disease burden underscores the need for a deeper investigation into the mechanisms driving CRC initiation and progression to enhance therapeutic strategies.

CRC is a complex genetic disease, with many genes influencing its onset and progression. Mutation in the Adenomatous polyposis coli (*APC*) tumor suppressor gene occurs in over 80% of sporadic colorectal adenomas, and germline mutations in the *APC* gene result in familial adenomatous polyposis (FAP) syndrome [2,3]. *APC* mutation leads to enhanced Wnt signaling, a key driver of CRC development [4]. Other frequent mutations include inactivation of tumor suppressors such as p53 and TGF-β, as well as oncogenic mutations in K-Ras, all of which are associated with increased tumor aggressiveness [5]. In addition to these canonical pathways, dysregulation of other signaling pathways also contributes to CRC initiation and progression, including those regulated by Rho-GTPases [6].

Rho-GTPases are small G-proteins belonging to the Ras superfamily that serve as key regulators of actin cytoskeleton dynamics. These proteins play pivotal roles in numerous cell functions, including proliferation, apoptosis, survival, cell adhesion, and migration [7]. Given their central role in these processes, it is not surprising that dysregulated Rho-GTPase signaling is implicated in cancer initiation and progression [8]. Among the Rho-GTPases, the most extensively studied members, RhoA, Rac1, and Cdc42, exhibit diverse roles in CRC. Increased Rac1 and Cdc42 expression and activation have been observed in human colorectal cancer samples, correlating with disease progression and poor prognosis [9,10,11]. Importantly, Rac1 contributes to CRC resistance to targeted therapies [12,13]. In the context of *APC* mutations, intestinal tumor cells activate Cdc42 to promote survival and facilitate microadenoma progression and, consequently, ablation of this GTPase attenuates the tumorigenicity in the Apc^Min/+^ mouse model of intestinal cancer [14]. In contrast to Rac1 and Cdc42, reduced RhoA expression has been associated with poor prognosis of colon cancer patients [15]. Furthermore, inactivation of RhoA increased tumor formation and progression in Apc^Min/+^ mice [16]. These findings highlight the complex, context-dependent roles of Rho-GTPases in CRC biology.

In addition to altered expression, various mechanisms contribute to the dysregulation of Rho-GTPase signaling in tumors [17,18], with a prominent role played by Rho-GTPase regulatory proteins. Rho-GTPases function as molecular switches, cycling between an inactive GDP-bound state and an active GTP-bound state. This process is tightly regulated by three types of regulatory proteins: Guanine nucleotide Exchange Factors (GEFs), GTPase-activating proteins (GAPs), and Guanine Dissociation Inhibitors (GDIs). GEFs activate Rho proteins by facilitating the exchange of GDP for GTP while GAPs and RhoGDIs serve as inhibitors [19]. Despite the substantial number of GEFs and GAPs (82 and 66, respectively), relatively few studies have explored their roles in CRC. Among the Rho-GEFs, Tiam1, βPix, GEF-H1, ArhGEF25, Asef, Asef2, and Vav3 have been investigated in human samples and/or animal models. These studies demonstrate that either overexpression or activation of these proteins favors colon cancer cell proliferation, migration, and invasion [20,21,22,23]. The information is even more limited in GAPs. Existing studies have primarily focused on the expression patterns of Rho-GAPs in CRC patients; however, in vivo investigations addressing the mechanisms by which Rho-GAPs regulate CRC development remain scarce [24].

In this study, we investigated the role of the Rac1-specific GAP β2-chimaerin in intestinal tumorigenesis using the Apc^Min/+^ mouse model. β2-chimaerin is a product of the *CHN2* gene, which also encodes the testis-specific β1-chimaerin and two other minor transcripts [25,26,27]. β2-chimaerin has demonstrated roles in regulating cell adhesion and migration [28,29,30], proliferation [28,31], T-cell activation [32], and insulin signaling [33]. Deregulation of the *CHN2* gene has been associated with various human pathologies, including cancers, where β2-chimaerin functions as a tumor suppressor [30,34]. In samples from colorectal cancer patients, the *CHN2* gene is hypermethylated compared to controls, which correlates with β2-chimaerin downregulation [35]. Notably, consistent hypermethylation of the *CHN2* gene has also been observed in small bowel adenocarcinoma [36], suggesting that β2-chimaerin downregulation may contribute to the incidence and progression of gastrointestinal tumors.

In the present study, we provide the first in vivo evidence that β2-chimaerin plays a tumor-suppressive role in intestinal tumorigenesis. Using β2-chimaerin knockout (KO) mice crossed with the ApcM^in/+^ model, we demonstrate that β2-chimaerin deficiency promotes colonic polyp growth, revealing a previously unrecognized role for this Rac1-specific GAP in colorectal cancer progression. Furthermore, we provide evidence for a role of β2-chimaerin in controlling proliferation as the molecular mechanism underlying this effect. Given the limited understanding of how Rho-GTPase regulatory proteins influence colorectal cancer, our findings expand current knowledge by establishing β2-chimaerin as a modulator of tumor growth in the colon. This study provides new insights into the molecular mechanisms of CRC and highlights β2-chimaerin as a potential target for further investigation in colorectal cancer therapy.

## 2. Results

### 2.1. β2-Chimaerin Deficiency Does Not Affect Polyp Development in the Small Intestine of Apc^Min/+^ Mice

**Figure 1:**
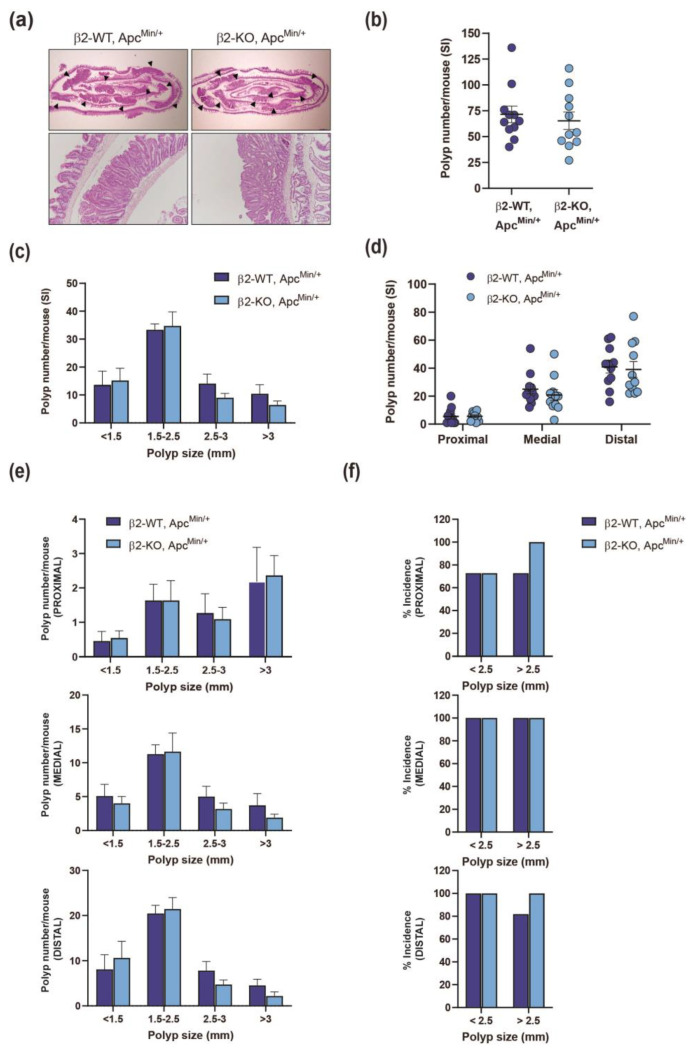
Effect of β2-chimaerin deletion on small intestine polyp formation in Apc^Min/+^ mice: β2-WT, Apc^Min/+^ and β2-KO, Apc^Min/+^ (*n* = 11/group) were sacrificed 4 months after birth, and small intestines were isolated and analyzed; (**a**) H&E staining of small intestine “Swiss roll” (upper panels). Polyps are marked with arrowheads. A higher magnification of a representative polyp is shown in the lower panels; (**b**) number of polyps/mouse in the SI (**c**) size distribution of polyps; (**d**) number of polyps/mouse in the proximal, middle, and distal portions of the SI; (**e**) size distribution in the proximal, middle, and distal sections of the SI; (**f**) % incidence of small (<2.5) or large (>2.5) polyps in the proximal, middle, and distal SI. Results are shown as mean ± SEM. SI, small intestine.

To investigate the role of β2-chimaerin in intestinal tumorigenesis, we generated compound mutant mice by crossing β2-chimaerin KO mice, generated by gene trapping [30], with Apc^Min/+^ mice. We evaluated the effect of β2-chimaerin deficiency on intestinal adenoma formation at 4 months of age, a time point when Apc^Min/+^ mice typically develop adenomatous polyps, while avoiding severe illness that manifests around 6 months [37]. (Figure 1).

The compound β2-KO, Apc^Min/+^ mice developed normally and exhibited similar body weight at the time of sacrifice compared to β2-WT, Apc^Min/+^ mice (24.1 ± 1.1 g vs. 25.9 ± 0.8 g, respectively). As expected, polyps predominantly developed in the small intestine in both genotypes, consistent with prior reports [38]. The histologic appearance of the neoplastic lesions in mice of both genotypes was similar among the two groups, with no evidence of submucosal invasion (Figure 1a). The total number of polyps in the small intestine was statistically indistinguishable between β2-KO, Apc^Min/+^ and β2-WT, Apc^Min/+^ mice (71.5 ± 8.1 vs. 66.4 ± 8.7, respectively, *p* = 0.6) (Figure 1b). Small intestine lengths were also similar between the groups (32.1 ± 0.8 for β2-WT, Apc^Min/+^ and 32.9 ± 0.6 cm for β2-KO, Apc^Min/+^). To determine whether β2-chimaerin deficiency influences polyp size distribution, we categorized polyps into four groups based on their diameter (<1.5, 1.5–2.5, 2.5–3, and >3 mm). We found no significant difference in size distribution between genotypes (Figure 1c).

The distribution of polyps in the small intestine of Apc^Min/+^ mice is known to be non-uniform. To investigate whether β2-chimaerin deficiency influences this pattern, we evaluated the number of polyps in the proximal, medial, and distal sections of the small intestine. The number of polyps was similar across the three sections in both β2-WT, Apc^Min/+^ and β2-KO, Apc^Min/+^ mice, with more polyps developed in the distal portion of the intestine as reported [38] (Figure 1d). We next analyzed polyp size distribution within each intestinal segment and found no significant differences between the genotypes (Figure 1e). Finally, we compared the incidence of smaller polyps (<2.5 mm) and larger polyps (>2.5 mm) in each segment. Both genotypes showed a high percentage of mice developing polyps of any size. Interestingly, β2-KO, Apc^Min/+^ mice exhibited a slightly higher incidence of larger polyps in the proximal (100% vs. 73%) and distal (100% vs. 82%) segments of the intestine compared to β2-WT; Apc^Min/+^ mice (Figure 1f).

Overall, this analysis indicates that β2-chimaerin deficiency does not have a substantial impact on the formation or growth of Apc^Min/+^-induced polyps in the small intestine.

### 2.2. β2-Chimaerin Deficiency Increases the Incidence of Large Colonic Polyps in Apc^Min/+^ Mice and Is Significantly Associated with Poor Prognosis of Colon Cancer Patients

**Figure 2:**
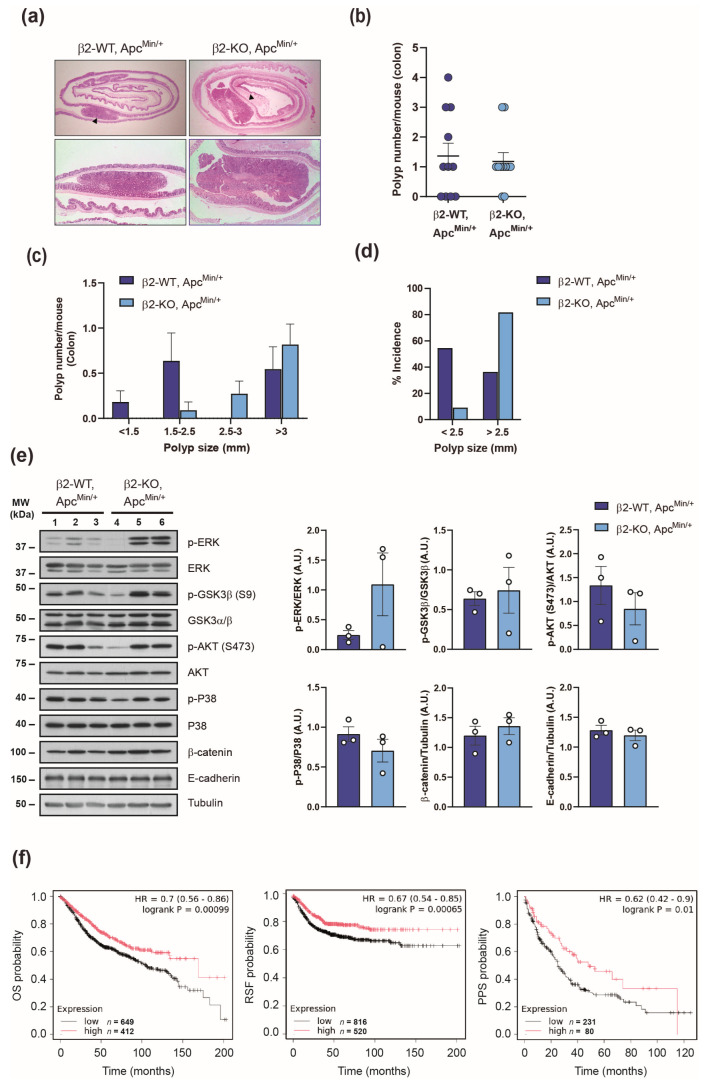
Effect of β2-chimaerin deletion on colorectal cancer: (**a**–**e**) Effect of β2-chimaerin deletion on colonic polyp formation in Apc^Min/+^ mice. β2-WT, Apc^Min/+^ and β2-KO, Apc^Min/+^ (*n* = 11/group), were sacrificed 4 months after birth, and colons were isolated and analyzed; (**a**) H&E staining of colorectal “Swiss roll” (**upper** panels), with polyps marked with arrowheads. A representative polyp is shown in the lower panels; (**b**) scatter plots showing the number of polyps/mouse of each genotype; (**c**) size distribution of polyps; (**d**) % incidence of small (<2.5) or large (>2.5) polyps. Results are shown as mean ± SEM; (**e**) Western blot analysis of the expression and phosphorylation status of the indicated proteins in homogenates from large (>2.5) colonic polyps from mice of the indicated genotypes (*n* = 3). Densitometric analyses are shown in the histograms. P-ERK, p-GSK3β, p-AKT, and p-P38 levels were normalized to the corresponding total protein (*p* = 0.25, *p* = 0.75, *p* = 0.40, and *p* = 0.30, respectively; Student’s *t*-test). β-catenin and E-cadherin protein levels were normalized to tubulin (*p* = 0.49 and *p* = 0.50, respectively; Student’s *t*-test). Results are shown as mean ± SEM; (**f**) Kaplan–Meier plots of overall survival (OS), relapse-free survival (RFS), and post-progression survival (PPS) of colon cancer patients stratified by expression of the *CHN2* gene; high (red) or low (black). Analysis was performed with the Kaplan–Meier Plotter, https://kmplot.com (accessed on 21 October 2024). Statistical significance was assessed by the log-rank test. The probe set for *CHN2* was 213385_at.

The Apc^Min/+^ mouse model is widely recognized for studying colorectal cancer, although fewer polyps develop in the colon compared to the small intestine. To determine whether β2-chimaerin deficiency influences colonic tumorigenesis, we examined polyp formation in the colons of β2-WT, Apc^Min/+^ and β2-KO, Apc^Min/+^ mice (Figure 2). At 4 months of age, mice of both genotypes developed colonic polyps with similar histological features (Figure 2a). The number of colonic polyps was equivalent in β2-WT, Apc^Min/+^ and β2-KO, Apc^Min/+^ (Figure 2b), and no significant difference was observed in colon length (6.8 ± 0.4 cm vs. 6.7 ± 0.4 cm, respectively).

Size-distribution analysis revealed that colonic polyps in β2-KO, Apc^Min/+^ were larger than those of β2-WT, Apc^Min/+^ mice, with a trend toward significance for polyps measuring 2.5–3 mm (*p* = 0.08) (Figure 2c).

In terms of the incidence, 63.6% of β2-WT, Apc^Min/+^ mice developed colonic polyps, compared to 81.5% of β2-KO, Apc^Min/+^ mice. Notably, 81.8% of β2-KO, Apc^Min/+^ developed tumors larger than 2.5 mm, compared to only 36.4% of control animals. Conversely, smaller polyps (<2.5 mm) were more prevalent in β2-WT, Apc^Min/+^ mice (54.5%) than in β2-KO, Apc^Min/+^ (9%) (Figure 2d). These results revealed that there is a switch to develop larger colonic polyps in Apc^Min/+^ mice lacking β2-chimaerin, which suggests that deficiency of this protein promotes polyp growth.

To explore the mechanisms underlying the increased growth of colonic polyps observed in β2-KO; Apc^Min/+^ mice, we examined the impact of β2-chimaerin ablation on signaling pathways relevant for colorectal tumorigenesis [39]. To this end, we performed Western blot analysis in lysates from large polyps (>2.5 mm) isolated from the colons of both genotypes at 4 months of age (Figure 2e). Polyps from β2-KO, Apc^Min/+^ mice clearly exhibited elevated levels of phosphorylated ERK, with approximately a fivefold increase compared to control polyps. Of note, this effect was not observed in polyps from the small intestine where ERK phosphorylation remained unchanged regardless of β2-chimaerin expression (Figure S1). Phosphorylation of glycogen synthase kinase 3β (GSK3β) was modestly increased, whereas levels of phosphorylated P38α or AKT were either unchanged or slightly reduced compared to controls. We would like to note that these results may underestimate the extent of phosphorylation due to abnormally low levels in one sample from the β2-KO, Apc^Min/+^ group, which affected statistical significance. However, the enhanced ERK phosphorylation in the KO samples remains clear and highly significant. Additionally, no notable differences were observed in the total levels of β-catenin or E-cadherin in polyps lacking β2-chimaerin. Collectively, these findings suggest that the loss of β2-chimaerin enhances proliferative signaling pathways, particularly via ERK activation, thereby promoting increased polyp growth.

To assess the relevance of our experimental findings in human colon cancer, we evaluated the prognostic value of the β2-chimaerin gene (*CHN2*) expression using a large clinical microarray database of colon cancer patients via the Kaplan–Meier plotter tool [40]. This analysis revealed that low *CHN2* expression in unstratified colon cancer patients significantly correlated with reduced overall survival (*p* < 0.001), relapse-free survival (*p* < 0.001), and post-progression survival (*p* < 0.05) (Figure 2f). These findings support a protective role of β2-chimaerin in colon cancer pathogenesis, consistent with a tumor-suppressive function of this protein.

## 3. Discussion

Dysregulation of Rho-GTPases is a key contributor to the development and progression of intestinal tumors. However, the specific roles of the many regulatory proteins that control Rho-GTPase activity remain insufficiently understood. In this study, we present the first in vivo evidence for a role of the Rac1-specific GAP β2-chimaerin in Apc-driven colon carcinogenesis.

To evaluate the impact of β2-chimaerin downregulation, we utilized the Apc^Min/+^ mouse model, which is widely recognized *to* resemble human colon cancer. This model effectively mimics both sporadic colorectal cancer, which has a high prevalence of APC mutation, and familial adenomatous polyposis (FAP), caused by APC loss [38]. Our results revealed that β2-chimaerin deletion increases the incidence and growth of colonic polyps, an effect likely through the regulation of ERK activation. These results are consistent with previous reports showing increased proliferation upon β2-chimaerin downregulation in epithelial cells [28] and inhibition of the ERK pathway when β2-chimaerin is overexpressed [31,41,42]. Notably, β2-chimaerin deletion did not enhance ERK activation in the small intestine, which may explain the lack of effect on polyp growth in this region.

The precise molecular mechanisms through which β2-chimaerin exerts these effects require further investigation. The selective effect of β2-chimaerin in the colon could be due to a differential expression of either β2-chimaerin or its targets along different regions of the intestine. Alternatively, β2-chimaerin may interact differently with Wnt pathway components depending on the intestinal segment. Similar region-specific effects on intestinal tumorigenesis have been observed for other proteins, such as PPARγ and KRas, whose tumorigenic roles are influenced by differences in expression patterns and molecular interactions across the intestine [43,44].

Since β2-chimaerin is known to regulate ERK via Rac1 activation [28,31], we hypothesize that increased Rac1 activity in polyps from β2-KO, Apc^Min/+^ mice underlies the observed enhanced activation of ERK. This hypothesis aligns with the well-established role of Rac1 in intestinal tumorigenesis following APC loss [45,46,47]. Supporting this notion, deletion of various Rac1-GEFs in the Apc^Min/+^ mice has been shown to reduce proliferation, which correlates with diminished Rac1 activity [22,48]. Furthermore, decreased expression of miR-142-3p has been associated with colorectal tumorigenesis via Rac1-ERK signaling [49].

The β2-chimaerin effect on ERK activation occurs downstream of the epithelial growth factor receptor (EGFR) [28,41]. *Apc* mutation is accompanied by increased EGFR expression and activity in the tumors, which explains the efficacy of EGFR-targeting therapeutics in the treatment of colorectal cancer patients of specific molecular subtypes [50,51,52]. Consequently, the deletion of β2-chimaerin in Apc^Min/+^ mice may further enhance EGFR signaling, leading to increased polyp growth.

Beyond ERK, we hypothesized that β2-chimaerin could also influence colon carcinogenesis through its role in regulating GSK3β. Recent findings have identified β2-chimaerin as a mediator of AKT-dependent GSK3β phosphorylation in response to insulin [33]. GSK3β is also a key component of the canonical Wnt/β-catenin, where it regulates β-catenin phosphorylation, targeting this protein for proteasomal degradation. In Apc^Min/+^ this pathway is upregulated, leading to β-catenin accumulation and increased transcription of β-catenin-mediated target genes that are essential for cell proliferation and CRC progression [4]. We hypothesized that ablation of β2-chimaerin in Apc^Min/+^ polyps might enhance GSK3β phosphorylation, thereby inactivating GSK3β and contributing to further β-catenin accumulation. However, our analysis showed that GSK3β phosphorylation and β-catenin levels remained almost unchanged in the absence of β2-chimaerin, suggesting that β2-chimaerin does not influence this axis in Apc-driven tumorigenesis. Additionally, a role of β2-chimaerin in the regulation of E-cadherin has been reported to influence breast cancer progression [30]. However, this function does not appear to play a role in Apc-driven tumorigenesis, as E-cadherin levels were unaffected by β2-chimaerin ablation.

In summary, this study shows in vivo that β2-chimaerin downregulation contributes to colon cancer, aligning with bioinformatics analyses that associate low expression of this protein with poor prognosis in patients. These findings expand on previous research on the tumor-suppressive functions of β2-chimaerin while also revealing key differences in its mechanisms of action, which influence distinct signaling pathways depending on upstream receptors. This underscores the complex, context-dependent roles of Rho-GTPases and their regulators in CRC biology.

## 4. Materials and Methods

### 4.1. Mice

All mice were housed at the Animal Research Facility of the University of Salamanca and the University of Valladolid. All animal care and protocols were reviewed and approved by the Institutional Animal Care and Use Committee and complied with the European Community directive 2010/63/EU.

The β2-chimaerin knockouts were obtained from Lexicon Genetics (Woodland, TX, USA). These mice were generated by gene-trap insertion in the β2-chimaerin gene (*Chn2*) and were described before [30]. Mice were originally in a mixed C57Bl/6/129/SvEvBrd background and were backcrossed to the C57Bl/6 background for 4 generations. C57BL/6J-Apc^Min/+^ mice were from the Jackson Laboratory (Bar Harbor, ME, USA). To generate β2-chimaerin-deficient Apc^Min/+^ mice, homozygous *Chn2*−/− mice were crossed with Apc^Min/+^ mice to generate double heterozygous mice that were then intercrossed to generate two cohorts: *Chn2*+/+ Apc^Min/+^ (referred to as β2-WT, Apc^Min/+^) and *Chn2*−/−, Apc^Min/+^ (referred to as β2-KO, Apc^Min/+^). All offspring were genotyped by PCR of tail DNA. Apc^Min/+^ genotyping has been described [38], and *Chn2* genotyping was performed according to Lexicon Genetics’s recommendations. All experiments were performed on age-matched (4-month-old) male mice.

### 4.2. Mouse Intestinal Tumor Analysis

Tumor scoring was performed as described [53]. At 4 months of age, β2-WT Apc^Min/+^ and β2-KO Apc^Min/+^ male mice were sacrificed, and their small intestine and colon were extracted and washed with PBS. The small intestine was divided into three equal segments (proximal, middle, and distal), and the colon was kept as a whole. Intestine segments were spread over 3 mm paper and fixed in 10% buffered formalin for 24 h at RT. 10% fixed intestines were stained with 0.5% methylene blue in distilled water to facilitate identification of small tumors. Macroscopic intestinal tumors were identified under a stereo microscope (Leica Ez4HD) (Leica Microsystems, Heerbrugg, Switzerland). The maximum polyp diameter was measured using the LAS EZ Imaging software (V2.0.0). The number and diameter of polyps in each of the four intestinal segments were recorded. The total body weight and length of the small intestine and colon were also measured at the terminal point. After tumor scoring, “Swiss rolls” of each intestinal section were embedded in paraffin, sectioned at 4 µm, and subjected to hematoxylin and eosin (H&E) staining.

### 4.3. Western Blot Assay

Intestinal polyps (mm) were weighted and homogenized in a lysis buffer containing 20 mM Tris–HCl (pH 7.4), 150 mM NaCl, 1% Triton X-100, 0.5 mM EDTA, 1 mM Na_3_VO_4_, 1 mM NaF, 1 mM DTT, and a mixture of protease inhibitors (Cømplete, Roche Molecular Biochemicals, Mannheim, Germany), using a Polytron tissue homogenizer (IIA-Ultra-Turrax T8) (IKA Werke GmbH & Co. KG, Staufen, Germany). After 10 min on ice, lysates were centrifuged at 12,000× *g* for 10 min at 4 °C to remove cell debris. Supernatants were collected, and protein content was quantified using the Bradford method. Equivalent amounts of protein were resolved by SDS-PAGE and processed by immunoblotting analysis [30,54].

The following primary antibodies were used: antibodies against p-ERK (Thr202/Tyr204) (#9101), ERK (#9102), p-AKT (Ser473) (#4060), AKT (#9272), p-GSK3β (Ser9) (#5558), β-Catenin (D10A8) (#8480), and p-P38 (Thr180/Tyr182) (#9211) were from Cell Signaling Technology (Danvers, MA, USA); GSK-3α/β (#VMA00342) was from BioRad (Hercules, CA, USA); P38 (sc-535) was from Santa Cruz Biotechnology (Dallas, TX, USA); E-cadherin (#610181) was from BD Transduction Laboratories (Crystal Lake, CA, USA); and Tubulin (#CP-06) was from Oncogene (Boston, MA, USA). Immunoblot–derived signals were quantified using Quantity One-1D image analysis 4.5 software (Bio-Rad).

### 4.4. Clinical Dataset Analysis

The online tool Kaplan–Meier Plotter https://kmplot.com (accessed on 21 October 2024). was used to explore the relation between β2-chimaerin (*CHN2*) expression and overall survival (OS), relapse-free survival (RFS), and post-progression survival (PPS) of unstratified colon cancer patients. Data from 1336 colon cancer patients retrieved from 11 GEO (Gene Expression Omnibus) databases were used in this analysis based on the updated 2024 version [40]. *p*-values are calculated with the log-rank test. In all studies, the probe set for *CHN2* was 213385_at.

### 4.5. Statistical Analyses

Statistical analyses were performed using GraphPad Prism 8.0 (GraphPad Software, Inc., Boston, MA, USA). Data are presented as mean ± SEM. Comparisons between the two groups were carried out using the two-tailed unpaired Student’s *t*-test. Differences were considered significant at *p* < 0.05.

## Data Availability

The data presented in this study are available on request from the corresponding author due to privacy.

## Supplementary Materials

The following supporting information can be downloaded at: https://www.mdpi.com/article/10.3390/molecules30040824/s1, Figure S1: β2-chimaerin deletion does not affect ERK activation in small intestine polyps.
