# Supplementary material for: β2-Chimaerin Deficiency Favors Polyp Growth in the Colon of ApcMin/+ Mice
Source: Molecules. 2025 Feb 11;30(4):824. doi: 10.3390/molecules30040824 (PMC11858732; doi:10.3390/molecules30040824)
Supplement: Supplementary file 1 [file molecules-30-00824-s001.zip › molecules-3377679-supplementary.pdf]

β2-chimaerin deficiency favors polyp growth in the colon of $Apc^{Min/+}$ mice

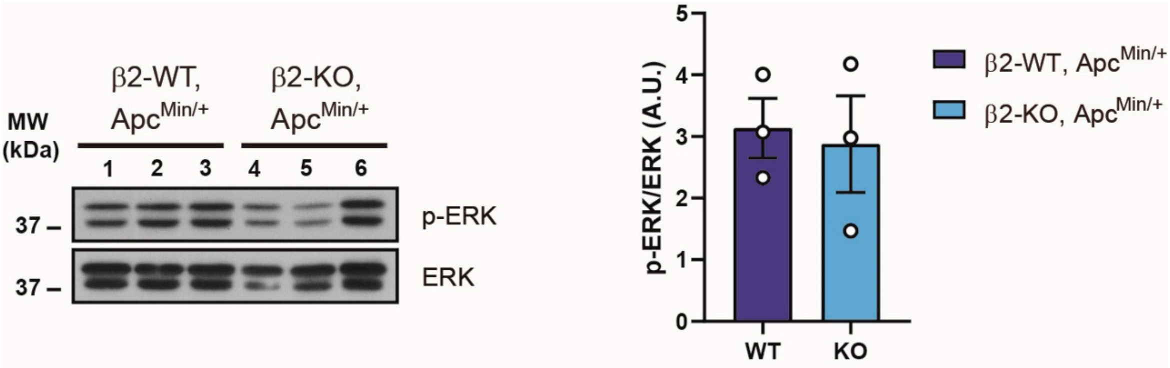

Figure S1. β2-chimaerin deletion does not affect ERK activation in small intestine polyps. Western blot analysis of the expression and phosphorylation status of ERK in homogenates from large (>2.5) small intestine polyps from mice of the indicated genotypes (n=3) (same mice than in Figure 2e). Densitometric analyses are shown in the histograms. P-ERK levels was normalized to the corresponding total protein (p = 0.79, Student's t-test). Results are shown as mean ± SEM.
